# Foxc1 is required by pericytes during fetal brain angiogenesis

**DOI:** 10.1242/bio.20135009

**Published:** 2013-05-20

**Authors:** Julie A. Siegenthaler, Youngshik Choe, Katelin P. Patterson, Ivy Hsieh, Dan Li, Shou-Ching Jaminet, Richard Daneman, Tsutomu Kume, Eric J. Huang, Samuel J. Pleasure

**Affiliations:** 1 Department of Neurology, Programs in Neuroscience and Developmental Biology, Institute for Regenerative Medicine, UC San Francisco, San Francisco, CA 94158, USA; 2 Department of Pathology, UC San Francisco, San Francisco, CA 94143, USA; 3 VA Medical Center, San Francisco, CA 94121, USA; 4 Beth Israel Deaconess Medical Center, Department of Pathology, Harvard School of Medicine, Boston, MA 02215, USA; 5 Department of Anatomy, UC San Francisco, San Francisco, CA 94143, USA; 6 Feinberg Cardiovascular Research Institute, Northwestern University School of Medicine, Chicago, IL 60611, USA; ‡ Present address: Department of Pediatrics, Denver-Anschutz Medical Campus, University of Colorado, Aurora, CO 80045, USA

**Keywords:** Angiogenesis, Blood brain barrier, foxc1, Neurovascular development, Pericyte

## Abstract

Brain pericytes play a critical role in blood vessel stability and blood–brain barrier maturation. Despite this, how brain pericytes function in these different capacities is only beginning to be understood. Here we show that the forkhead transcription factor Foxc1 is expressed by brain pericytes during development and is critical for pericyte regulation of vascular development in the fetal brain. Conditional deletion of *Foxc1* from pericytes and vascular smooth muscle cells leads to late-gestation cerebral micro-hemorrhages as well as pericyte and endothelial cell hyperplasia due to increased proliferation of both cell types. Conditional *Foxc1* mutants do not have widespread defects in BBB maturation, though focal breakdown of BBB integrity is observed in large, dysplastic vessels. qPCR profiling of brain microvessels isolated from conditional mutants showed alterations in pericyte-expressed proteoglycans while other genes previously implicated in pericyte–endothelial cell interactions were unchanged. Collectively these data point towards an important role for Foxc1 in certain brain pericyte functions (e.g. vessel morphogenesis) but not others (e.g. barriergenesis).

## Introduction

During neural development appropriate growth and maturation of the brain vasculature is essential to meet increasing metabolic needs and to establish a barrier between the brain and potentially damaging agents circulating in the blood. An important step in brain angiogenesis is the association of pericytes with forming brain capillaries. Responding to platelet-derived growth factor-B (PDGFB) released by endothelial cells (ECs), PDGF receptor-β (PDGFrβ) expressing pericytes home towards and spread along the vessel surface (for a review, see Armulik et al., 2011). Deletion of either *PDGFB* or *PDGFrβ* results in reduction in pericyte investment of the vasculature in several organs, including the brain and retina (Bjarnegård et al., 2004; Hellström et al., 2001; Lindahl et al., 1997). In the fetal brain, loss of pericyte coverage does not overtly impact vessel patterning, however, there are significantly more endothelial cells (ECs) per vessel (hyperplasia), and vessels are susceptible to hemorrhage (Hellström et al., 2001; Lindahl et al., 1997). Moreover, pericytes play an essential role in the formation and maintenance of the blood brain barrier (BBB) and establishment of the neurovascular unit. Pericyte-deficient mutants (*PDGFB*-null or hypomorph and *PDGFrβ*-null) display systemic brain vascular leak and, postnatally, fail to establish proper polarity of the astrocyte endfeet (Armulik et al., 2010; Daneman et al., 2010b). The defects in the fetal brain vasculature of pericyte-deficient mutants provide compelling evidence that pericytes are critical for stabilization and maturation of the neural vascular plexus.

While it is clear that brain pericytes have critical functions in both the fetal and adult brain vasculature (for reviews, see (Bergers and Song, 2005; Gaengel et al., 2009; Winkler et al., 2011), there is less known about how they function in these different capacities. *In vitro*, pericytes inhibit EC proliferation in a cell contact-dependent manner (Orlidge and D'Amore, 1987) and this is due at least in part to activation of transforming growth factor-β1 (TGFβ1) (Antonelli-Orlidge et al., 1989). Pericyte-derived TGFβ1 also plays a role in BBB-induction in pericyte–EC co-culture systems (Dohgu et al., 2005). Pericytes are a potentially important source of vascular endothelial growth factor (VEGF); retinal pericytes express VEGF and pericyte derived-VEGF is an important pro-survival cue for cultured ECs (Darland et al., 2003). Pericytes are also a purported source of angiopoietin-1 (Sundberg et al., 2002), the agonist for the endothelial Tie2 receptor critical for vessel stabilization during development (Suri et al., 1996; Suri et al., 1998; Thurston et al., 1999). Collectively this evidence suggests that cell–cell contact and production of diffusible ligands are a major part of how brain pericytes function *in vivo*. Up to this point, however, no transcription factors with the potential to influence multiple signaling pathways in pericytes have been identified.

Foxc1, a forkhead transcription factor expressed by a variety of mesenchymal tissues, is particularly important for development of head structures. *Foxc1* hypomorphs or null mouse mutants have moderate to severe defects in eye, craniofacial and brain development (Aldinger et al., 2009; Kume et al., 2000; Siegenthaler et al., 2009; Smith et al., 2000; Zarbalis et al., 2007; Kume et al., 1998). *Foxc1*-null mutants have also been reported to have considerable brain hemorrhaging toward the end of gestation that is largely limited to the forebrain region (Grüneberg, 1943; Kume et al., 1998). Expression of Foxc1 has been localized to the brain endothelium (Kume et al., 1998) and we have previously shown that Foxc1 is expressed in perivascular cells in the brain and meninges (Zarbalis et al., 2007). Here we show more definitively that Foxc1 is expressed by brain pericytes and that it is essential for normal pericyte function in the developing brain. Specifically, conditional deletion of *Foxc1* from pericytes and vascular smooth muscle cells (vSMCs) leads to alterations in both brain pericyte and EC proliferation and the appearance of late-gestation brain micro-hemorrhage.

## Results

### Foxc1 is expressed by pericytes and vSMCs in the fetal brain

**Fig. 1.**
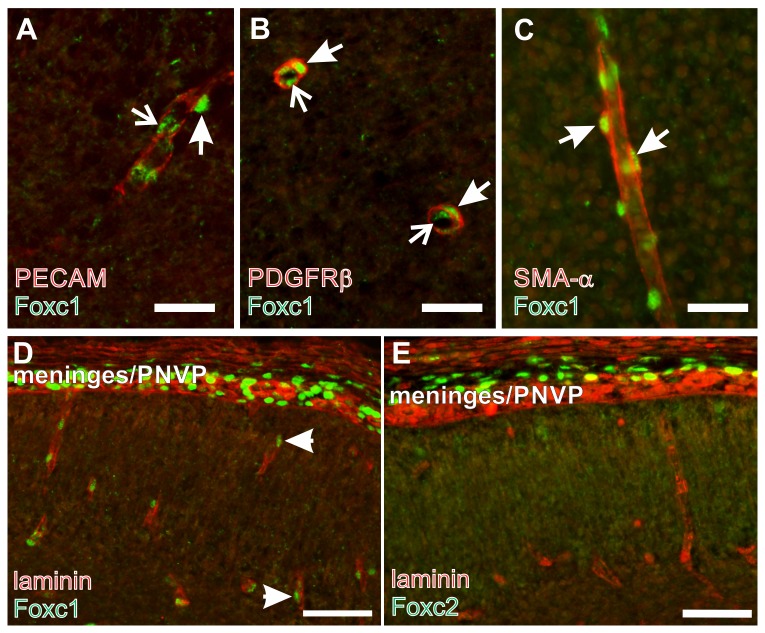
Foxc1 is expressed by brain pericytes. (**A**) Foxc1 (green) and PECAM (red) co-immunolabeling of a cerebral vessel at E14.5. Arrowhead indicates Foxc1+ cells immediately adjacent to the PECAM+ vessel. Arrows indicate Foxc1+ cell in the inner lumen of the PECAM+ vessel. (**B**) Arrowheads indicate Foxc1 (green) and PDGFrβ (red; B) or SMA-α (red; **C**) co-labeled pericytes around the outside of cerebral blood vessels. Arrows indicate Foxc1+ cell in the inner lumen of the vessel cross-section. (**D**,**E**) Foxc1 (D) and Foxc2 (E) in the meninges and adjacent cortex at E14.5. Laminin (red) labels cerebral vessels. Scale bars: 25 µm (A–C); 50 µm (D,E).

There are numerous Foxc1+ cells immediately adjacent to large vessels in the meninges and associated with small vessels in the brain parenchyma, which we suspected were pericytes (Zarbalis et al., 2007). Foxc1 is expressed by the meningeal cells and, like forebrain pericytes, these cells are derived from the cranial neural crest (Etchevers et al., 2001). On the other hand, Foxc1 and its close homologue Foxc2 are important for early EC specification and vascular development in the early embryo (De Val et al., 2008; Seo et al., 2006). To definitively identify the Foxc1 expressing cell type in the brain vasculature, we used markers of ECs (PECAM) and pericyte/vSMC (PDGFrβ, smooth muscle actin-α) in conjunction with Foxc1 immunostaining. At E14.5, Foxc1+ cells were found on the outside of PECAM+ blood vessels in the brain (Fig. 1A), the characteristic location for pericytes. In addition, Foxc1+ nuclei were observed within the vessel which is consistent with expression by endothelial cells (Fig. 1A). Pericyte and vSMCs labeled by PDGFrβ and smooth muscle actin-α, respectively, had Foxc1+ nuclei (Fig. 1B,C), indicating that Foxc1 is expressed by brain pericytes and vSMCs. As with PECAM, we observed Foxc1+ cells within the vessel lumen of blood vessels, presumable ECs, outlined with PDGFrβ (Fig. 1B). Foxc2, a close homologue of Foxc1, is frequently co-expressed with Foxc1 and double-KO studies indicate that these two genes have redundant functions developmentally (Kume et al., 2000; Kume et al., 2001; Seo et al., 2006). Immunostaining for Foxc2 revealed expression in some cells in the meninges but, unlike Foxc1, there was no expression associated with the vasculature in the brain (Fig. 1D,E). This suggests that Foxc2 cannot compensate for Foxc1 activity in brain pericytes and vSMCs, thus making it possible that loss of Foxc1 alone could negatively impact pericyte function.

### Increased pericyte proliferation in *Foxc1* mutant brains

**Fig. 2.**
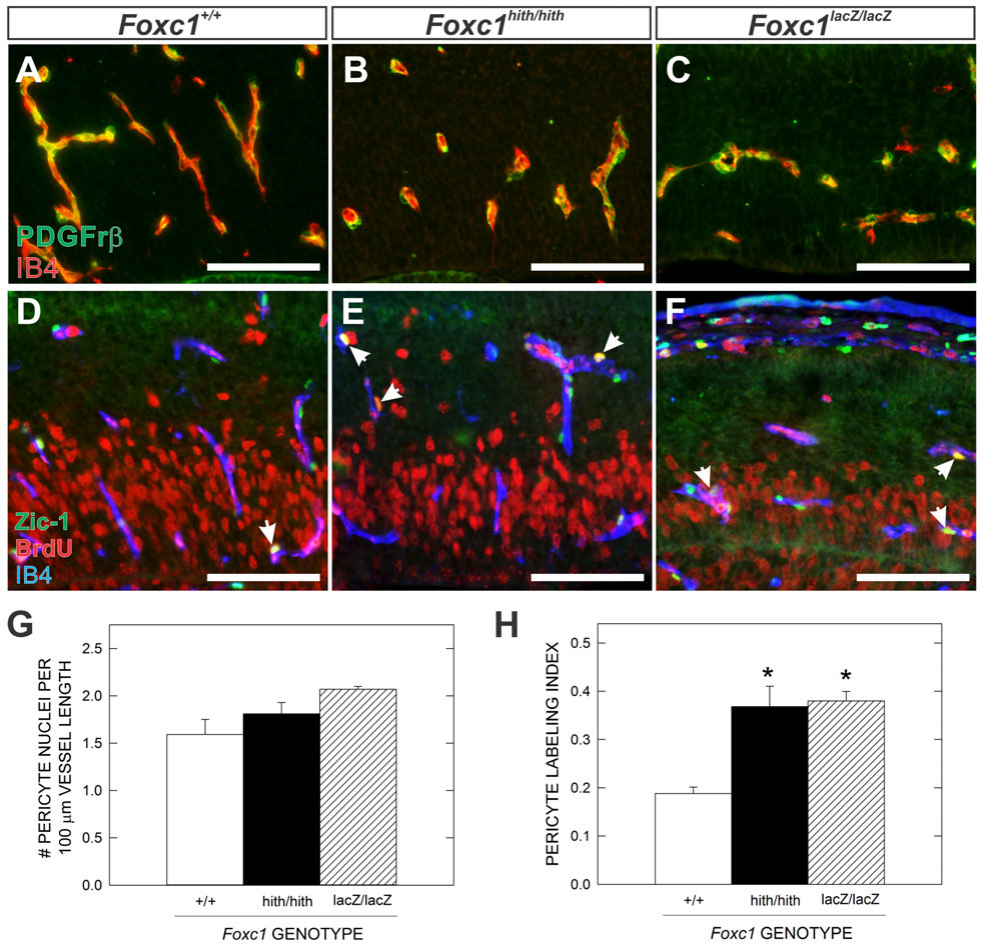
Altered brain pericyte proliferation in *Foxc1* mutants. (**A–C**) PDGFrβ (green) and IB4 (red) immunofluorescence highlight pericyte cell bodies in the cortices of E14.5 WT, *Foxc1^h/h^* and *Foxc1^l/l^*. (**D–F**) Triple immunofluorescence for Zic1 (pericyte nuclei; green), BrdU (red) and IB4 (blue) highlight proliferating (Zic1+/BrdU+; arrows) pericytes in the E14.5 cerebral vasculature of WT, *Foxc1^h/h^* and *Foxc1^l/l^* embryos. (**G**) Graph depicting quantification of Zic1+ pericyte nuclei/vessel length in all three genotypes at E14.5. (**H**) Graph depicting quantification of pericyte proliferation (labeling index) at E14.5. Scale bars: 100 µm. Means represent analysis of three independent litters for each mutant genotype (*n* = 3). Asterisks indicate a statistically significance difference from WT (*P*<0.05). Error bars depict ±SEM.

**Fig. 3.**
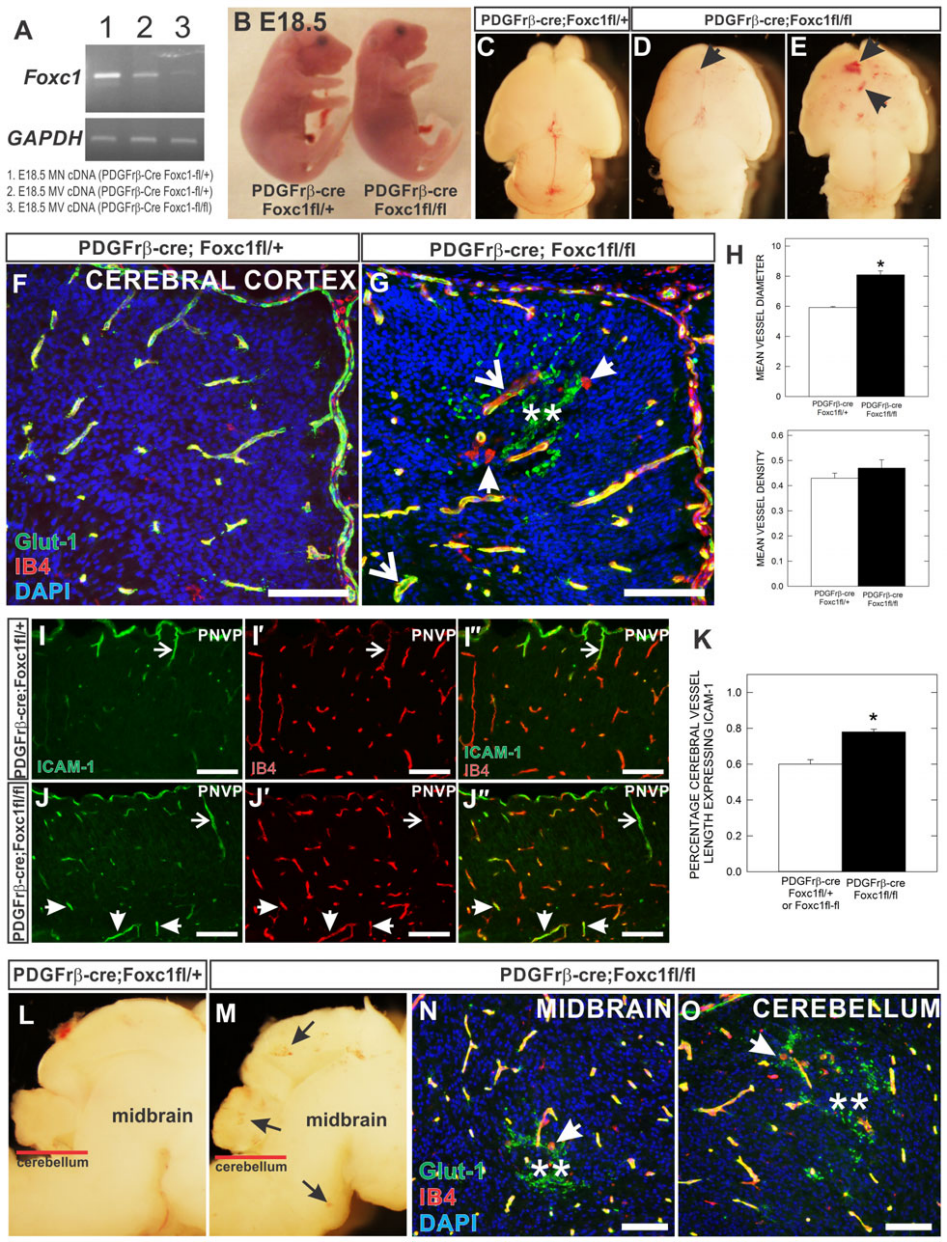
Vascular defects and brain micro-hemorrhage in pericyte conditional *Foxc1* mutants. (**A**) RT-PCR of *Foxc1* transcript from three RNA sources: (1) E18.5 *PDGFrb-cre*; *Foxc1fl-+* meninges (2) E18.5 microvessels from *PDGFrb-cre*; *Foxc1fl-+* brain and (3) E18.5 microvessels from *PDGFrb-cre*; *Foxc1fl-fl* brain. Housekeeping gene *GAPDH* serves as internal control. (**B**) Whole embryo images of E18.5 *PDGFrβ-cre*; *Foxc1fl-fl* mutant and *PDGFrb-cre*; *Foxc1fl-+* littermate. (**C–E**) Dorsal view of E18.5 *PDGFrβ-cre*; *Foxc1fl-+* (C) and *PDGFrβ-cre*; *Foxc1fl-fl* mutant (D,E) brains. Arrows indicate hemorrhage within the cerebral cortex. (**F**,**G**) Glut-1 (green), Ib4 (red) and DAPI (blue) stained cortical sections of E18.5 *PDGFrβ-cre*; *Foxc1fl-fl* mutant and *PDGFrb-cre*; *Foxc1fl-+* animals. (**H**) Graphs depicting quantification of average cerebral vessel diameter (top) and cerebral vessel density (bottom) in E18.5 *PDGFrβ-cre*; *Foxc1fl-fl* mutant and *PDGFrβ-cre*; *Foxc1fl-+* animals. Means represent analysis from three separate litters (*n* = 3). (**I**,**J**) ICAM-1 and IB4 immunofluorescence of E18.5 *PDGFrβ-cre*; *Foxc1fl-+* and *PDGFrβ-cre*; *Foxc1fl-fl* cortices. Arrowheads indicate superficial ICAM-1+ cerebral vessels descending from the perineural vascular plexus (PNVP). Arrows indicate deeper, ICAM-1+ vessels in *PDGFrβ-cre*; *Foxc1fl-fl* mutant cortices. (**K**) Quantification of percentage of ICAM+ cerebral vessels in control and mutant samples analyzed from three independent litters (*n* = 3). (**L**,**M**) Sagittal view of midbrain and cerebellum of E18.5 *PDGFrβ-cre*; *Foxc1fl-fl* mutant and *PDGFrb-cre*; *Foxc1fl-+* brains. Arrows indicate hemorrhage sites in *PDGFrβ-cre*; *Foxc1fl-fl* sample. (**N**,**O**) Glut-1 (green), Ib4 (red), DAPI (blue) immunofluorescence at the level of the midbrain (N) and cerebellum (O) in E18.5 *PDGFrβ-cre*; *Foxc1fl-fl* mutant brains. In G,N,O, arrowheads indicate amoeboid morphology of Ib4+ activated microglia; arrows indicate dilated/dysplastic vessels; asterisks indicate red blood cells in the neural parenchyma. Scale bars: 100 µm. Asterisks in graph indicate a statistically significant difference from *PDGFrβ-cre*; *Foxc1fl-+* samples (*P*<0.05). Error bars depict ±SEM.

Reduction in pericyte coverage of blood vessels leads to brain vascular defects and micro-hemorrhages (Hellström et al., 2001). *Foxc1* null mice (*Foxc1-LacZ*) and *Foxc1* hypomorph mutants (*Foxc1-hith*) have developmental brain hemorrhages (Kume et al., 1998; Zarbalis et al., 2007). While these vascular defects are more severe than what would be expected with pericyte loss, it is possible that alterations in pericyte coverage might contribute to the *Foxc1* brain vascular phenotype. Using PDGFrβ expression to visualize pericyte sheaths around cerebral vessels at E14.5, it appeared that pericyte coverage was comparable between wildtype (WT), *Foxc1^h/h^* and *Foxc1^l/l^* mutants (Fig. 2A–C). Quantification of pericyte coverage using a nuclear marker of brain pericytes and meningeal cells, Zic-1 (Daneman et al., 2010b; Inoue et al., 2008), revealed an increase in pericyte nuclei per vessel length in *Foxc1* mutants though it was not significantly different from WT (Fig. 2D–G). Interestingly, pericyte proliferation (as determined by BrdU incorporation) was significantly (*P*<0.05) increased in both types of *Foxc1* mutants (Fig. 3D–F,H). Changes in pericyte proliferation may reflect an inability to properly respond to differentiation signals controlling pericyte expansion and suggests that although the pericytes are present in *Foxc1* mutants, they may not be functioning properly.

### Focal brain hemorrhage and increased pericyte number in pericyte conditional *Foxc1* mutants

Since *Foxc1* mutants have significant defects in both brain and vascular development, it is difficult to determine from these mutants the cell-autonomous role of Foxc1 in brain pericytes or ECs. Conditional deletion of *Foxc1* from ECs causes no overt brain vascular phenotype in the adult animal (Hayashi and Kume, 2008). Thus, Foxc1 is unlikely to have a significant, cell-autonomous role in brain ECs. To address whether Foxc1 is important for brain pericyte function during development, we used the *PDGFrβ-cre* line, previously shown to recombine all pericytes and vSMCs in the brain by E10.5 (Stenzel et al., 2009), and a *Foxcl-flox* allele (Hayashi and Kume, 2008; Sasman et al., 2012) to conditionally ablate *Foxc1* from this cell population. To confirm knockdown of *Foxc1* expression in brain pericytes, we performed RT-PCR for *Foxc1* transcript on cDNA from control meninges (high expression of Foxc1) or microvessels (contain both ECs and pericytes) isolated from control brains or *PDGFrβ-cre*; *Foxc1fl-fl* brains (Fig. 3A). *Foxc1* transcript is appreciable lower in *PDGFrβ-cre*; *Foxc1fl-fl* microvessels; the residual expression is likely *Foxc1* in ECs.

*PDGFrβ-cre;Foxc1fl-fl* mutants were similar in size to littermate controls at E18.5 (Fig. 3B) but died at birth. Thus our analysis of the brain vasculature was limited to the prenatal period. At E18.5, *PDGFrβ-cre;Foxc1fl-fl* brains had small, focal hemorrhages in the cerebral cortex not observed in a littermate control (Fig. 3C–E). Although all *PDGFrβ;Foxc1fl-fl* examined displayed vascular defects in the forebrain, the number and size of obvious hemorrhages varied from mild (Fig. 3D) to more severe (Fig. 3E). Glut-1 immunostaining of E18.5 cortices revealed a relatively normal vascular pattern in *PDGFrβ-cre;Foxc1fl-fl* but abnormally enlarged vessels and Glut-1+ red blood cells in the neural parenchyma indicating hemorrhage distinguished the mutants from littermate controls (Fig. 3F,G). Indeed, mean cerebral vessel diameter was significantly increased in *PDGFrβ-cre;Foxc1fl-fl* mutants but cerebral vessel density was unaltered (Fig. 3H). Isolectin B4 (IB4) staining, which labels both blood vessels and microglia, highlighted the amoeboid morphology of activated microglia around the hemorrhage site in the *PDGFrβ-cre;Foxc1fl-fl* cortex (Fig. 3G); activated microglia are characteristic of an inflammatory response, presumably caused by the hemorrhage. We also observed upregulation of endothelial ICAM-1 in E18.5 *PDGFrβ-cre;Foxc1fl-fl* cerebral vessels (Fig. 3I,J) which is associated with endothelial cell activation and increased inflammatory cell trafficking (Dietrich, 2002). Quantification of ICAM-1 expression revealed that a significantly higher percentage of cerebral vessel length expressed ICAM-1 in the *PDGFrβ-cre;Foxc1fl-fl* mutants than in control at E18.5 (Fig. 3K). Enlarged vessels and activated microglia were seen as early as E15.5 and E16.5 in *PDGFrβ-cre;Foxc1fl-fl* mutant cortices (supplementary material Fig. S1). We also observed small hemorrhages in the midbrain and cerebellum of *PDGFrβ-cre;Foxc1fl-fl* mutants at E18.5 (Fig. 3L–O), though the size and number of the micro-hemorrhages was less frequent than in rostral brain structures.

**Fig. 4.**
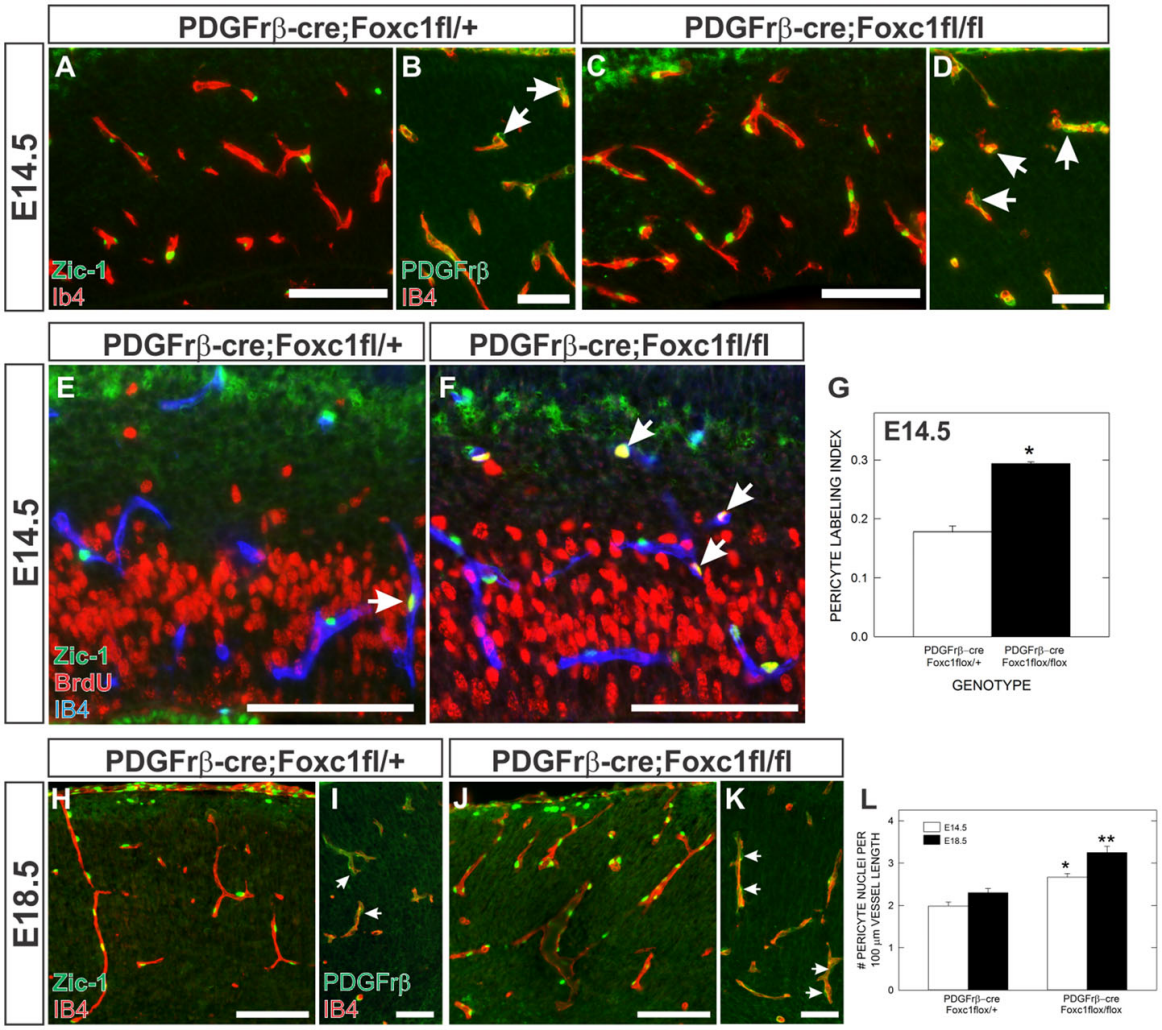
Pericyte conditional *Foxc1* mutants have increased pericyte proliferation and density in the cerebral vasculature. (**A**,**C**) Zic1 (green) and IB4 (red) co-immunofluorescence in the cortices of *PDGFrβ-cre*; *Foxc1fl-fl* mutant and *PDGFrβ-cre*; *Foxc1fl-+* brains at E14.5. (**B**,**D**) PDGFrβ (green) and IB4 (red) co-immunofluorescence in the cortices of *PDGFrβ-cre*; *Foxc1fl-fl* mutant and *PDGFrβ-cre*; *Foxc1fl-+* brains at E14.5. Arrowheads indicate PDGFrβ+ pericytes. (**E**,**F**) Zic1 (green), BrdU (red), and IB4 (blue) triple immunofluorescence of E14.5 *PDGFrβ-cre*; *Foxc1fl-fl* mutant and *PDGFrβ-cre*; *Foxc1fl-+* cerebral cortex. Arrows indicate Zic1+/BrdU+ pericytes. (**G**) Graph depicts quantification of Zic1+ pericyte proliferation (labeling index) in the cerebral vasculature at E14.5. (**H**,**J**) Zic1 (green) and IB4 (red) co-immunofluorescence in the cortices of *PDGFrβ-cre*; *Foxc1fl-fl* mutant and *PDGFrβ-cre*; *Foxc1fl-+* brains at E18.5. (**I**,**K**) PDGFrβ (green) and IB4 (red) co-immunofluorescence in the cortices of *PDGFrβ-cre*; *Foxc1fl-fl* mutant and *PDGFrβ-cre*; *Foxc1fl-+* brains at E18.5. Arrowheads indicate PDGFrβ+ pericytes. (**L**) Graph depicts pericyte density (Zic1+ pericyte nuclei/vessel length) in the cerebral vasculature at E14.5 and E18.5 in *PDGFrβ-cre*; *Foxc1fl-fl* mutant and *PDGFrβ-cre*; *Foxc1fl-+* animals. Asterisks indicate a statistically significance difference from *PDGFrβ-cre*; *Foxc1fl-+* samples (*P*<0.05). Analysis of pericyte proliferation and density was performed on control and mutant brains from three separate litters at each time point (*n* = 3). Error bars depict ±SEM. Scale bars in A,C,E,F,H,J: 100 µm. Scale bars in B,D,I,K: 50 µm.

We next used Zic-1 and PDGFrβ immunolabeling along with BrdU incorporation to determine whether pericyte density and proliferation in the cerebral vasculature was altered in *PDGFrβ-cre;Foxc1fl-fl* mutants at E14.5. We chose to look at this developmental stage because it is prior to observable hemorrhage and vascular dysplasia. At E14.5, more Zic-1+ pericyte nuclei were associated with cortical blood vessels in *PDGFrβ-cre;Foxc1fl-fl* mutants and many of these were also BrdU+ (Fig. 4A,C,E,F). Indeed, both pericyte proliferation and pericyte nuclei per vessel length were significantly increased in *PDGFrβ-cre;Foxc1fl-fl* mutants compared to littermate controls at E14.5 (Fig. 4G,L). Even more pericytes covered cerebral vessels in *PDGFrβ-cre;Foxc1fl-fl* mutants at E18.5 (Fig. 4H,J) demonstrated by a significant increase in pericyte nuclei per vessel length from E14.5 to E18.5 in the mutants (Fig. 4L). This progressive increase in pericyte number is likely due to dysregulated pericyte proliferation in the absence of Foxc1. As with *Foxc1* mutants, PDGFrβ immunolabeling did not reveal any significant changes in vessel coverage by pericytes though, consistent with Zic-1 anlysis, there were more pericyte cell bodies in the *PDGFrβ-cre;Foxc1fl-fl* mutants at both E14.5 (Fig. 4B,C) and E18.5 (Fig. 4I,K).

During the course of our analyses of the vascular phenotype in *PDGFrβ-cre;Foxc1fl-fl* mutant brain, we noted a moderate lengthening of the cerebral neuroepithelium, first observed at E15.5 and also present at E17.5 (supplementary material Fig. S2A–D). This phenotype appeared to be a much milder version of the cortical phenotype we previously described in *Foxc1^h/h^* and *Foxc1^l/l^* mutants in which absence of the forebrain meninges and meningeal-derived signals leads to defects in cortical neurogenesis (Siegenthaler et al., 2009). This, in conjunction with the perinatal lethality of *PDGFrβ-cre*; *Foxc1fl-fl* mutants, suggested that *PDGFrβ-cre* activity is not specific for the pericyte and vSMC population and is active in other tissues that express Foxc1. Examination of *PDGFrβ-cre* recombinase activity using the *Rosa26-YFP* reporter line revealed that cre activity was limited to pericytes and vSMCs in the PNVP and in the head and brain vasculature at E12.5 (supplementary material Fig. S2E–G), but by E13.5 there was also recombination in the facial and calvarial mesenchyme, and in a large portion of the meninges (supplementary material Fig. S2H,I). Given that Foxc1 is critical for development of the forebrain meninges (Siegenthaler et al., 2009; Vivatbutsiri et al., 2008) we next determined whether the meninges were affected by the later recombination of *Foxc1* using Zic-1 immunolabeling. The number of Zic-1+ meningeal cells was normal at E14.5 in *PDGFrβ-cre;Foxc1fl-fl* mutants, however, by E16.5 there was an obvious decrease in Zic-1+ cells overlying the cortex (supplementary material Fig. S2J–M). Presumably, this mid-corticogenesis decline in meningeal coverage causes the cortical phenotype in *PDGFrβ-cre;Foxc1fl-fl* mutants, however, we do not believe that the meningeal defects and mild cortical defects contribute to the brain vascular phenotype in these mutants. This is supported by the fact that meninges loss and mild cortical defects in *PDGFrβ-cre;β-catenin-lof-lof* mutants (Choe et al., 2012) are not associated with cerebral vascular defects (supplementary material Fig. S3).

### Foxc1-deficient pericytes associate normally with hyperplastic cerebral blood vessels

**Fig. 5.**
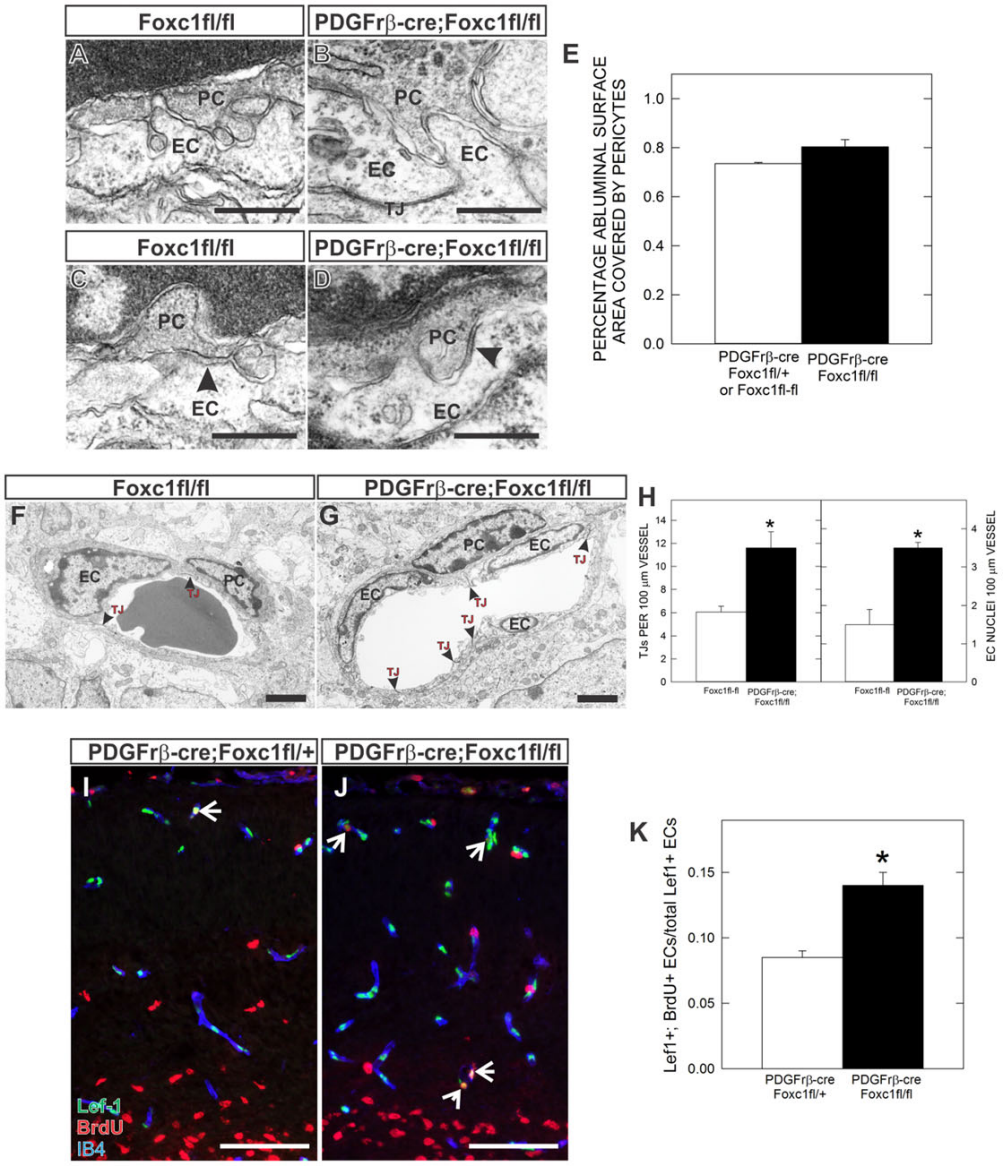
Ultrastructure analysis of pericyte–EC interactions reveals EC hyperplasia and increased EC cell proliferation in conditional *Foxc1* mutants. (**A–D**) High magnification image of contact points between EC and pericyte cell membranes highlight pericyte–EC interactions – “peg and socket” (A,B) and adhesion plaques (arrows in C,D) in cerebral vessels of *Foxc1fl-fl* and *PDGFrβ-cre*; *Foxc1fl-fl* mutant animals. (**E**) Graph depicting analysis of pericyte coverage of abluminal surface of cerebral blood vessels in E18.5 control and mutant samples. (**F**,**G**) EM images of cerebral vessel cross-sections highlighting increased EC nuclei and tight-juntions (red arrows) in *PDGFrβ-cre*; *Foxc1fl-fl* mutant cortices. (**H**) Graph depicting quantification of tight junction density (left) and EC nuclei density (right) from EM images of *Foxc1fl-fl* and *PDGFrβ-cre*; *Foxc1fl-fl* mutant animals. (**I**,**J**) Triple immunofluoresence for Lef-1 (green: ECs), BrdU (red) and IB4 (blue) in E16.5 *PDGFrβ-cre*; *Foxc1fl-+* and *PDGFrβ-cre*; *Foxc1fl-fl* cortices. Arrows indicates Lef-1+/BrdU+ ECs. (**K**) Graph depicts quantification of Lef-1+ EC proliferation (labeling index) in the cerebral vasculature at E16.5. PC: pericyte. EC: endothelial cell. TJ:tight junctions. Scale bars: 0.5 µm (A–D); 2 µm (F,G); 100 µm (I,J). Asterisks indicate a statistically significance difference between control and mutant samples (*P*<0.05). EM analysis was performed on images from three *PDGFrβ-cre*; *Foxc1fl-fl* samples (*n* = 3) and two control *Foxc1fl-fl* samples (*n* = 2). EC proliferation analysis was performed on control and mutant brains from two independent litters (*n* = 2). Error bars depict ±SEM.

Alterations in pericyte association with CNS blood vessels interfere with their ability to regulate vascular stability and BBB permeability (Li et al., 2011). Thus we used electron microscopy (EM) to evaluate pericyte–EC interactions in the cortices of E18.5 *PDGFrβ-cre;Foxc1fl-fl* mutants and littermate controls. Pericytes were always close to the abluminal surface of ECs in both control and *PDGFrβ-cre;Foxc1fl-fl* mutants (Fig. 5F,G). There was a small increase in the percentage of EC surface area covered by pericyte cell bodies and processes (Fig. 5E) though it was not statistically significant. This indicates that the increase in pericyte number does not result in abnormal distribution or pericyte process overlap. Importantly, we observed cytoplasmic interdigitation of pericytes and ECs (“peg-and-socket” junctional complexes) (Fig. 5A,B) and adhesion plaques (Fig. 5C,D) with similar frequency in both control and *PDGFrβ-cre;Foxc1fl-fl* mutants. Collectively this evidence indicates that, despite the increased numbers of pericytes, characteristic pericyte–EC interactions are overtly normal in *PDGFrβ-cre;Foxc1fl-fl* mutants.

The EM analysis also highlighted a significant increase in the number the EC nuclei in *PDGFrβ-cre;Foxc1fl-fl* mutant cerebral vessels, and subsequently, tight junctions connecting the supernumerary ECs (Fig. 5F–H). These changes are consistent with an increase in EC number within the cerebral blood vessels and may underlie the increased vessel size observed in these mutants. Pericytes are known to suppress EC cell proliferation *in vitro* (Orlidge and D'Amore, 1987) thus EC hyperplasia in *PDGFrβ-cre;Foxc1fl-fl* mutants may be caused by increased EC proliferation. To confirm this, we determined the proportion of proliferating ECs in control and mutant vessels at E16.5 using BrdU pulse labeling in conjunction with a nuclear marker of ECs, Lef-1. Lef-1 is a Wnt-regulated transcription factor, and, consistent with the role of Wnt signaling in the neurovasculature (Daneman et al., 2009; Stenman et al., 2008), it is expressed at high levels in all brain EC nuclei. Consistent with our analysis of vessels at the EM level, Lef-1+ EC nuclei were more numerous in *PDGFrβ-cre;Foxc1fl-fl* mutant cerebral vessels (Fig. 5I,J). Importantly, the proportion of BrdU+/Lef+ cells in mutant brains was significantly elevated (Fig. 5K), indicating that EC proliferation is increased and likely the main contributor to the hyperplastic vascular phenotype in these mutants.

### Limited BBB leakage in *PDGFrβ;Foxc1fl-fl* mutants

**Fig. 6.**
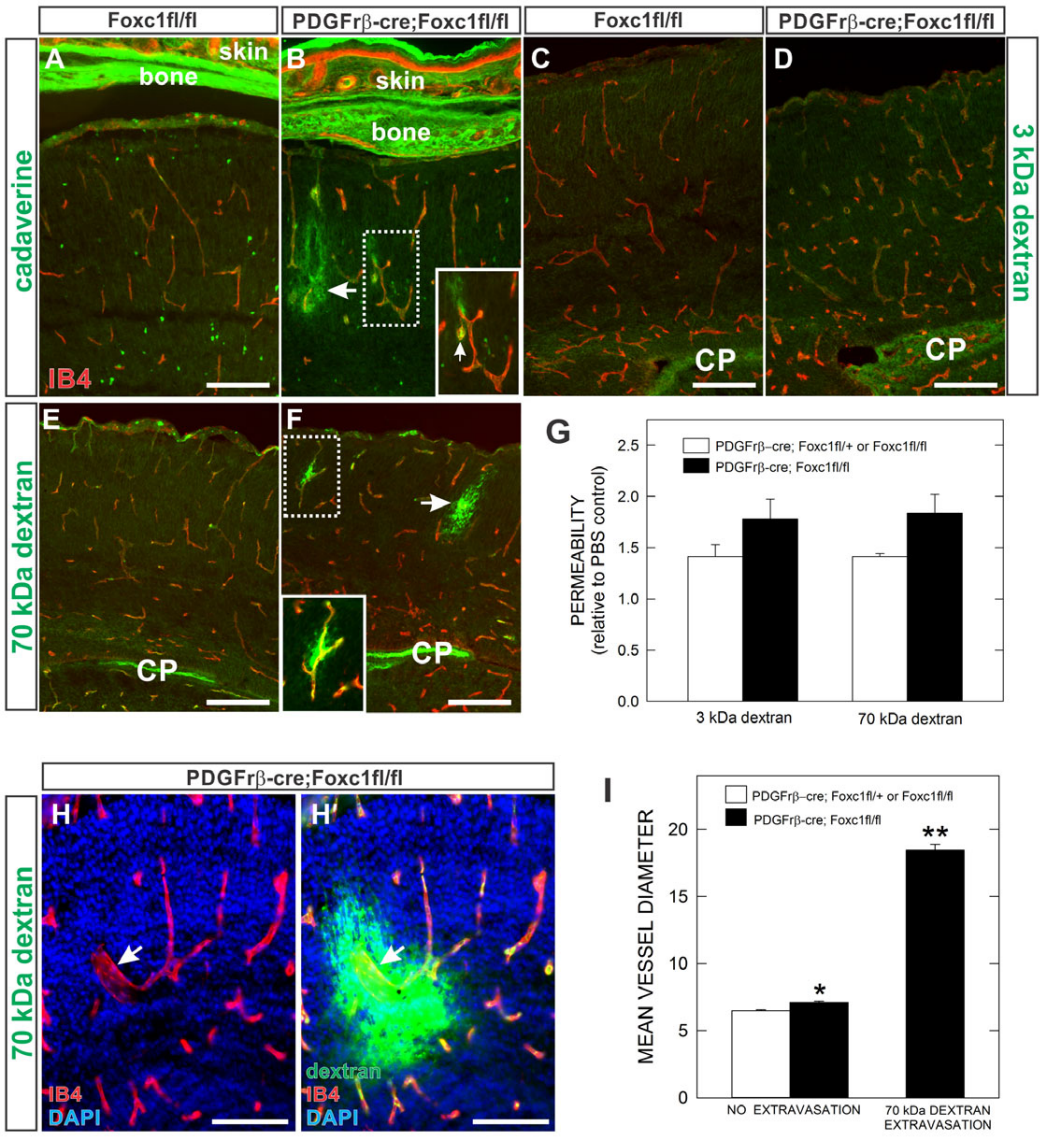
BBB integrity in pericyte conditional *Foxc1* mutants. (**A**,**B**) *Foxc1fl-fl* and *PDGFrβ-cre*; *Foxc1fl-fl* mutants injected with fluorophore-linked cadaverine tracer show strong labeling of bone and skin tissues where the tracer leaked out of fenestrated (non-barrier) vessels, but no significant leakage in brain tissue except for adjacent to a hemorrhage site in the *PDGFrβ-cre*; *Foxc1fl-fl* mutant brain (arrow). Magnified inset in (B) highlights tracer leak out of a vessel (arrow) and an adjacent activated, IB4+ microglia with amoeboid morphology (arrowhead). (**C–F**) Representative images of biotin-conjugated 3kDa (C,D) and 70 kDa dextran tracers (E,F) from E18.5 *Foxc1fl-fl* and *PDGFrβ-cre*; *Foxc1fl-fl* mutants cortices. Note tracer signal trapped in choroid plexus (CP). In F, magnified inset is of cerebral vessel with leakage of 70 kDa dextran tracer. (**G**) Graph depicts quantification of BBB permeability to 3 kDa and 70 kDa dextran tracers as measured by fluorescent intensity of cerebral tissue relative to PBS control. (**H**,**H′**) Representative large diameter cerebral vessel (Ib4+, red) in *PDGFrβ-cre*; *Foxc1fl-fl* mutant brain displaying vascular leak of 70 kDa tracer (green). (**I**) Graph depicting mean cerebral vessel diameter of vessels without and with 70 kDa tracer extravasation in control and mutant samples. Scale bars: 100 µm. Asterisks indicate a statistically significance difference from control samples (*P*<0.05). Analysis of BBB permeability was performed on three control and three mutant brains (*n* = 3). Error bars depict ±SEM.

Brain pericytes have long been thought to play roles in BBB formation and maintenance (Dente et al., 2001; Dohgu et al., 2005), however recent work showing severe defects in the integrity of the BBB in pericyte-deficient mutants provides the most compelling evidence to date (Armulik et al., 2010; Daneman et al., 2010b). To examine the BBB in *PDGFrβ-cre;Foxc1fl-fl* mutants, we assessed cerebral vascular permeability to large (70 kDa dextran) and small (3 kDa dextran and cadaverine: ∼1kDa) molecules at E18.5. *PDGFrβ-cre;Foxc1fl-fl* mutants did not display widespread permeability to dextrans or cadaverine but had focal extravasation of tracers, especially cadaverine (Fig. 6A,B) and 70 kDa dextran (Fig. 6E,F) in the vicinity of hemorrhages. Larger sites of tracer leakage were often accompanied by activated microglia (Fig. 6B), supporting the idea that tracer leak is associated with an active hemorrhage site. In other cases, extravasation of both 70 kDa dextran and cadaverine was limited to a small area immediately adjacent to a single vessel (Fig. 6B,F). Other than these areas of focal leakage, we did not detect significant vascular permeability to 70 kDa or 3 kDa dextran (Fig. 6G) nor was there widespread neuronal uptake of leaked cadaverine as was described previously in pericyte-deficient mutants (Fig. 6A,B) (Armulik et al., 2010).

Tracer extravasation was sometimes observed in small diameter vessels (Fig. 6B,F), though it was more common to observe tracer leakage in grossly enlarged vessels in *PDGFrβ-cre;Foxc1fl-fl* mutant brains (Fig. 6H). To quantify this, we determined the average vessel diameter of vessels with no leakage in control and mutant brains as well as the average vessel diameter of vessels with extravasation of 70 kDa dextran in mutant brains. There was a small but significant increase in vessel diameter in mutants with no 70 kDa leakage as compared to control (Fig. 6I). In contrast, the average vessel diameter of vessels with 70 kDa extravasation was substantially larger than no-leak vessels in either mutant or control (Fig. 6I). These data suggest that increased vessel size, likely caused by elevated EC proliferation, correlates with vessel instability.

### Expression profiling of pericyte conditional *Foxc1* mutant microvessels

**Fig. 7.**
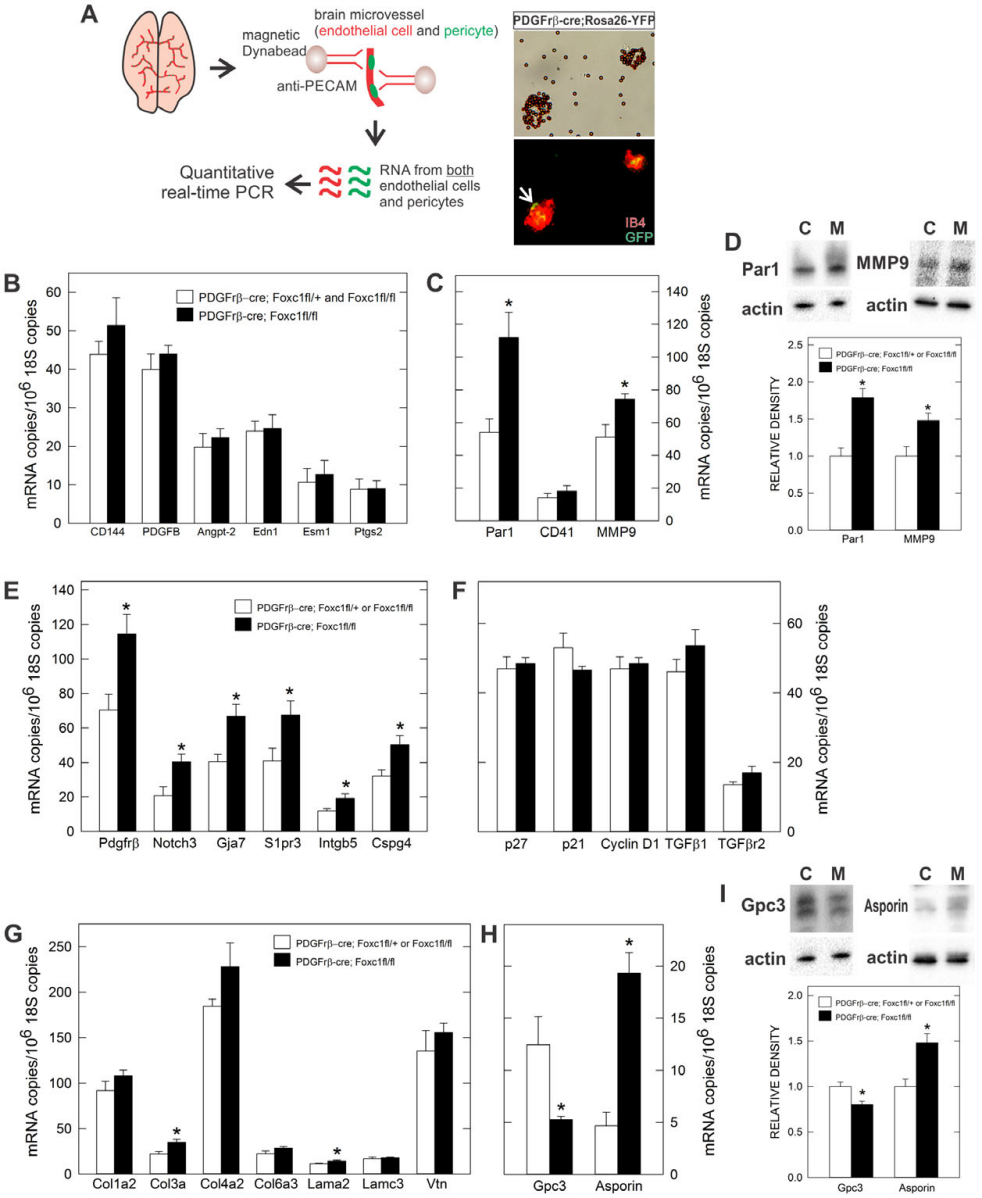
qPCR profiling of pericyte conditional *Foxc1* mutant brain microvessels. (**A**) Schematic of microvessel isolation from E18.5 brain and both brightfield (top) and fluorescence (bottom) image of microvessels following magnetic bead isolation. Arrow indicates recombined, GFP+ pericyte associated with IB4+ microvessel isolated from *PDGFrb-Cre*; *Rosa26-YFP* brain (**B**,**C**). Graphs depicting expression analysis of EC-expressed genes. (**D**) Immunoblots of Par1 and MMP9 on whole cortex lysate from E18.5 *PDGFrβ-cre*; *Foxc1fl-+ or Foxc1fl-fl* (control or “C”) and *PDGFrβ-cre*; *Foxc1fl-fl* (mutant or “M”). Graphs depict relative intensity of the Par1 or MMP9 band to control samples. (**E–I**) Graphs depicting expression analysis of pericyte-enriched genes (E), cell cycle genes and TGFβ signaling components (F), pericyte-enriched ECM genes (G), and pericyte-enriched proteoglycans (H). (I) Immunoblots of glypican-3 and asporin on whole cortex lysate from E18.5 *PDGFrβ-cre*; *Foxc1fl-+ or Foxc1fl-fl* (control) and *PDGFrβ-cre*; *Foxc1fl-fl* (mutant). Graphs depict relative intensity of the glypican-3 or asporin band to control samples. Asterisks indicate a statistically significance difference from control samples (*P*<0.05). qPCR analysis was performed on RNA isolated from six mutant (*n* = 6) and five control (*n* = 5) E18.5 brains. Immunoblot analysis was performed on lysates from three control and three mutant cortices (*n* = 3). Error bars depict ±SEM.

Microarray profiling of microvessels from pericyte-deficient mutants has generated both a comprehensive list of genes enriched in brain pericytes and revealed changes in endothelial cell gene expression consistent with increased vascular instability (Armulik et al., 2010; Bondjers et al., 2006; Daneman et al., 2010a; Daneman et al., 2010b). Using these published microarray data as well as the pericyte literature, we compiled a list of relevant pericyte and endothelial cell expressed genes to examine for changes in expression in microvessels isolated from pericyte conditional *Foxc1* mutants using quantitative real time-PCR. Microvessels, which contain both ECs and pericytes, were isolated from E18.5 control (*PDGFrβ-cre;Foxc1-fl/+* and *Foxc1fl/fl*) and *PDGFrβ-cre;Foxc1fl/fl* brains using PECAM-coated magnetic beads (Fig. 7A). We confirmed that both cell types were present by examining microvessels isolated from *PDGFrβ-cre/+;Foxc1-fl/+;Rosa26-YFP/+* brains; recombined, GFP+ pericytes were visibly associated with IB4+ blood vessels (Fig. 7A).

We first looked at expression of endothelial cell enriched genes, some of which were upregulated in pericyte-deficient microvessels and thus may be involved in the vascular defects observed in these mutants. Expression of endothelial cell-specific genes *CD144* (*VE-Cadherin*) and *PDGFB* were not significantly altered in *PDGFrβ-cre;Foxc1fl/fl* microvessels (Fig. 7B). Further, genes previously shown to be upregulated in pericyte-deficient brain microvessels and known to be involved in vascular homeostasis (*Angpt2*, *Edn1*, *Esm1*, and *Ptgs2*) were not significantly altered (Fig. 7B). However, two genes known to be expressed by ECs, *F2R* and *MMP-9*, were significantly upregulated in *PDGFrβ-cre;Foxc1fl/fl* microvessels at both the mRNA and protein levels (Fig. 7C,D). F2R, more commonly known as PAR-1, is a thrombin receptor that mediates thrombin-induced EC permeability after injury (Komarova et al., 2007). PAR-1 is also expressed by platelets, which could be present at greater numbers in pericyte conditional *Foxc1* mutants due to active hemorrhage sites, although we did not see increased expression of the platelet marker *CD41* (Fig. 7C). Since PAR-1 is also expressed by vSMCs, and potentially pericytes, it is possible this increase is due to expression in either ECs or vSMCs/pericytes. MMP9, through degradation of the ECM, can alter BBB permeability (Asahi et al., 2001) but is frequently increased after brain ischemia. Consequently, the increase in MMP-9 may be a result rather than the cause of the vascular leak. Nevertheless, elevated MMP-9 levels could exacerbate the existing vascular instability phenotype our mutants.

We next looked at expression of genes enriched in pericytes, classified as such by decreased expression in brain microvessels isolated from pericyte-deficient mice and expression analysis *in situ* (Armulik et al., 2010; Bondjers et al., 2006; Daneman et al., 2010b). We found that several pericyte-enriched genes (*PDGFrβ*, *Notch3*, *S1pr3*, *Intgβ5*, *Cspg4*, *Gja7*) were uniformly upregulated 1.6–1.7 fold compared to control (Fig. 7E), perhaps due to the fact that more pericytes are associated with blood vessels in pericyte conditional *Foxc1* mutants rather than increased expression on a cellular level. This is supported by the fact that we do not see an appreciable increase in PDGFrβ protein expression in individual brain pericytes (Fig. 4I,K). Cell proliferation defects in both pericytes and ECs point toward cell cycle regulators like p21, p27, and cyclin D1 as well as TGFβ signaling, which has been previously implicated in pericyte-mediated control of EC proliferation *in vitro* (Antonelli-Orlidge et al., 1989). We did not, however, detect significant changes in gene expression of these cell cycle regulators, TGFβ1 ligand or TGFβ receptor 2.

Extracellular matrix (ECM) proteins and proteoglycans, either membrane bound and within the ECM, contribute significantly to the rich, extracellular environment that encases blood vessels and pericytes. ECs and pericytes secrete the majority of the ECM proteins that make up the vascular basement membrane. Loss of ECM expression can significant affect brain vascular stability (Gould et al., 2005; Gustafsson et al., 2013) thus we determined whether ECM genes expressed by pericytes were altered in microvessels from *PDGFrβ-cre;Foxc1fl/fl* microvessels. A collagen subtype (*Col3a*) and a laminin subtype (*Lama2*) were significantly increased, though overall there were no major changes in laminin, collagen and vitronectin gene expression (Fig. 7G). Also, continuity of laminin and a collagen subtype, Col4a, protein expression along the vessel surface was not altered between control and mutant samples at E18.5 (supplementary material Fig. S4), indicating that ECM deposition and organization is not significantly affected. Collectively this suggests that the ECM-enriched vascular basement membrane is intact in *PDGFrβ-cre;Foxc1fl/fl* mutant brains. We did, however, identify two pericyte-expressed proteoglycans that showed notable changes in expression in the pericyte conditional *Foxc1* mutants. *Glypican-3* expression was downregulated 2-fold whereas *Asporin* was upregulated 4-fold in mutant microvessels (Fig. 7H). Changes in asporin and glypican-3 protein expression were verified at the protein level using immunoblots of whole brain lysates (Fig. 7I). Glypican-3, and to a lesser extent asporin, have established roles in modulating growth factor signaling pathways like TGFβ, Wnt, Sonic Hedgehog (Shh), BMP and FGF in part by sequestering ligands away from or chaperoning ligands to their cell surface receptors (Capurro et al., 2005; Capurro et al., 2008; Hartwig et al., 2005; Ikegawa, 2008; Stigliano et al., 2009). Altered composition of these pericyte-expressed proteoglycans in the absence of Foxc1 may alter growth factor signaling at the cell surface and, in this way, negatively impact pericyte and EC proliferation.

## Discussion

In this study we identified Foxc1 as a critical transcription factor regulating multiple aspects of pericyte function in the developing brain. Foxc1 is expressed by brain pericytes and vSMCs during development and late gestation pericyte-conditional *Foxc1* mutants display dysplastic vessels and micro-hemorrhages, most prominently in the cerebral cortex.

We believe that one of the key phenotypes in pericyte conditional *Foxc1* mutants is the increase in both pericyte and EC proliferation. For pericytes, this equates to a small (∼2 vs. ∼3 pericyte/100 µm vessel) but significant increase in pericyte number. Despite the increase in number, Foxc1-deficient pericytes associate normally with ECs and display overtly normal coverage of the vessel surface. This is an important observation in light of recent work showing that the brain hemorrhage and BBB defects in a cerebrovascular-specific knockdown of Smad4 are accompanied by pericyte detachment (Li et al., 2011). For ECs, it is unlikely that the increase in cell proliferation reflects an increase in angiogenic growth in the mutant vasculature, in large part because there is no observed increase in vessel density in pericyte conditional *Foxc1* mutants. Rather, we suspect that in the absence of the correct signal from the pericytes, there is a failure in EC quiescence that normally accompanies vessel maturation and stability. Ectopic cell division within a capillary likely causes an uneven increase in diameter along the vessel length, crowding of endothelial cell bodies and, potentially, destabilization of junctional contacts that must reorganize to incorporate the newly born cell. These structural changes may leave vessels vulnerable to the shear stress forces of blood flow and are likely a major causal factor for the micro-hemorrhages in our mutants. Indeed, vascular leak indicative of weak cell–cell contacts was most frequently observed in abnormally large diameter blood vessels in the conditional *Foxc1* mutants.

One major question is how Foxc1, when deleted in only pericytes and vSMCs using the *PDGFrβ-Cre*, can regulate cell proliferation in both pericytes and ECs. On the one hand, Foxc1 may directly regulate expression of genes involved in pericyte proliferation. Foxc1, downstream of TGFβ signaling, is important for growth suppression in several human cancer cell lines (Zhou et al., 2002), however, there is growing evidence that high expression of Foxc1 is correlated with increased breast cancer growth (Wang et al., 2012). Also, we observed no changes in TGFβ1 ligand expression or in expression of TGFβr2, the key signaling receptor for TGFβ ligands. Further, important cell cycle regulators were also unchanged in pericyte conditional *Foxc1* mutants. With no firmly established role for Foxc1 in regulating cell proliferation, one intriguing possibility comes from profiling data on microvessels isolated from pericyte conditional *Foxc1* mutants where we observed altered expression of pericyte-expressed proteoglycans asporin and glypican-3. Asporin is the predominant, non-collagen extracellular matrix protein in cartilage where it has been shown to bind to and block TGFβ1-activated signaling in chondrocytes (Nakajima et al., 2007). Glypican-3 is a heparin sulfate proteoglycan that is widely expressed in embryonic tissues where it has been shown to modulate several signaling pathways, including Sonic hedgehog, FGF and Wnt (Fico et al., 2011). Glypican-3 is membrane bound with a large extracellular domain that can interact with either the surface-bound receptors or ligands to modulate signaling “strength” either positively or negatively. The collective effect of glypican-3 on these pathways translates into growth inhibition. The extracellular space between the surface of a pericyte and EC is narrow and proteoglycans secreted by (e.g. asporin) or bound to the surface (e.g. glypican-3) of pericytes can potentially affect receptor-mediated signaling occurring at the surface of both cell types. Thus in the absence of Foxc1, brain pericytes fail to express appropriate levels of proteoglycans like asporin and glypican-3 and, in this way, may disrupt normal cell surface regulation of signaling pathways involved in cell proliferation.

Pericyte-conditional *Foxc1* mutants have similar phenotypic features as pericyte-deficient mutants, including large-diameter, hyperplastic cerebral vessels and vascular instability (Lindahl et al., 1997; Hellström et al., 2001). Unlike pericyte deficient mutants (Armulik et al., 2010; Daneman et al., 2010b), pericyte conditional *Foxc1* mutants do not display systemic permeability to large and small molecular weight tracers in the fetal brain. This suggests Foxc1 is not involved in pericyte-mediated BBB formation. Importantly, we did not observe evidence of increased endothelial trancytosis at the EM level, which is the probable mechanism underlying the increased BBB permeability in the pericyte-deficient mutants. Further, two genes implicated in pericyte-induced barriergenesis, *angiopoietin-1* and *angiopoietin*-2, are not significantly altered in our mutants. Pericyte–EC direct contact, which is intact in pericyte conditional *Foxc1* mutants, is also likely important for the ability of pericytes to limit BBB permeability. Indeed, pericyte–EC co-cultures in which the two cells types are in contact displayed a greater decrease in vascular permeability than non-contact co-cultures (Dohgu et al., 2005).

The late-gestation brain vascular defects and micro-hemorrhages in pericyte conditional *Foxc1* mutants are much milder that the severe cerebral hemorrhage observed in *Foxc1*-null mutant embryos at the same developmental stage (Kume et al., 1998). Though Foxc1 is expressed by brain ECs, severe vascular defects are not seen in EC-conditional *Foxc1* mutants (Hayashi and Kume, 2008). This suggests a non-cell autonomous contribution to the vascular phenotype in *Foxc1* mutant mice. Neocortical development is disrupted in *Foxc1* mutant mice due to increased self-renewing divisions by neural progenitors at the expense of neuron production (Siegenthaler et al., 2009). The result is a long, thin neocortex. Critical neuroangiogenic molecules like VEGF and Wnt ligands are produced, to varying degrees, by neural progenitors and post-mitotic neurons within the developing brain (Breier et al., 1992; Stenman et al., 2008). Thus the neural tissue likely creates angiogenic gradients that blood vessels use to properly grow within the developing brain. The disrupted forebrain architecture of *Foxc1* mutant mice may disturb the formation of these gradients, thus negatively impacting vascular growth. Retinoic acid, normally produced by the meninges but lacking in meninges-deficient *Foxc1* mutants, improves many of the neocortical defects in *Foxc1* mutant mice (Siegenthaler et al., 2009). In later gestation, non-retinoic acid exposed *Foxc1* mutant mice display severe cerebral hemorrhage whereas retinoic acid treated embryos do not, suggesting that the rescue of neocortical defects may also improve vascular development (J.A.S. and S.J.P., unpublished observations). Further analysis comparing retinoic acid rescued *Foxc1* mutants and pericyte conditional *Foxc1* mutants could prove useful is determining the relative contribution of pericyte dysfunction and defects in neocortical development to the vascular defects in *Foxc1*-null mice.

Recent work on CNS pericytes has revealed an important role for these cells in various CNS disease states, including as BBB gatekeepers warding off neurodegeneration (Bell et al., 2010; Bell et al., 2012) and as major participants in the formation in the fibrotic scar following spinal cord and stroke-induced brain injury (Göritz et al., 2011; Fernández-Klett et al., 2013). Given the variety of pericyte activities in the brain endothelium, dissecting how pericytes function in different capacities becomes somewhat problematic. The utility of the pericyte conditional *Foxc1* mutants is that the brain pericytes are present and functioning normally in some capacities (e.g. BBB permeability) but fail in others (e.g. vascular morphogenesis and stability). Thus, in conjunction with existing and future work on the pericyte deficient mutants, we hope to use pericyte conditional *Foxc1* mutants to connect pericyte-derived signals or properties with specific effects on the brain vasculature. Identifying the means by which pericytes ensure brain vascular integrity may provide new therapies for treating CNS diseases that involve vascular instability and disruption of the BBB.

## Materials and Methods

### Animals

Generation and genotyping of *Foxc1*-null and *Foxc1-hith* mice has been described previously (Zarbalis et al., 2007; Siegenthaler et al., 2009). *Foxc1-flox* mice were generated by T. Kume (Sasman et al., 2012) and genotyped using the following primers: 5′-ATTTTTTTTCCCCCTACAGCG-3′ and 5′-ATCTGTTAGTATCTCCGGGTA-3′. *PDGFrβ-cre* mice were generated by Ralf Adams at Cancer Research UK (Foo et al., 2006) and generously provided by Calvin Kuo (Stanford University). *PDGFrβ-cre* mice were maintained as heterozygotes and breeders or embryos were genotyped for the presence of the transgenic allele using standard *cre* primers. *Rosa26-YFP* mice were obtained from Jackson Laboratories (Bar Harbor, ME). To obtain mutant embryos, timed-matings were set up in the afternoon and females were examined for a vaginal plug the following morning. The day the plug was observed was counted as embryonic day (E) 0.5. All mice were housed in specific-pathogen-free facilities approved by AALAC and were handled in accordance with protocols approved by the UCSF Committee on Animal Research.

### Immunohistochemistry

Embryos (E12.5–E16.5) were collected and whole heads (E12.5–E14.5) or whole brains (≤E15.5) were fixed overnight in 4% paraformaldehyde. For E18.5, whole heads were fixed as described above or a transcardiac perfusion was performed with 3 ml PBS followed by 3 ml 4% paraformaldehyde. All tissues were cryoprotected with increasing sucrose concentrations and frozen in OCT. Tissue was cryosectioned in 12 µm increments. Immunohistochemistry was performed on tissue sections as previously described (Zarbalis et al., 2007) using the following antibodies: rabbit anti-Glut-1 1:500 (NeoMarkers/Thermo Scientific); mouse anti-BrdU 1:50 (BDBioscience); rabbit anti-Zic1 1:100 (Novus Biologicals); goat anti-Foxc1 1:300 (Novus Biologicals); goat anti-Foxc2 1:300 (Novus Biologicals); rabbit anti-PECAM 1:200 (NeoMarkers/Thermo Scientific); rabbit anti-Fli1 1:200 (NeoMarkers/Thermo Scientific); rabbit anti-Lef-1 1:100 (Cell Signaling Technology); mouse anti-SMAα 1:100 (Sigma-Aldrich); rabbit anti-PDGFrβ 1:200 (Cell Signaling Technology); rat anti-ICAM-1 1:200 (Abcam); chicken anti-GFP 1:500 (Invitrogen); rat anti-PDGFrα 1:200 (BDBioscience); rabbit anti-laminin 1:100 (Sigma). Following incubation with primary antibodies, sections were incubated with appropriate Alexafluor-conjugated secondary antibodies (Invitrogen), Alexafluor 633-conjugated isolectin-B4 (Invitrogen), and DAPI (Invitrogen). For Zic1, Lef-1, Foxc1, and Foxc2 antibodies, immunostaining was performed using the Tyramide System Amplification (TSA) Kit (Invitrogen) per manufacturer's instructions. All immunofluorescent and brightfield images were captured using a Retiga CCD-cooled camera and associated QCapture Pro software (QImaging Surrey, BC Canada).

### Electron microscopy and ultrastructure analysis

E18.5 *PDGFrβ-cre*; *Foxc1fl-fl* (*n* = 3) and *Foxc1fl-fl* littermate controls (*n* = 2) were perfused with 4% paraformaldehyde, embedded in Histogel (Thermo Scientific) and 400 µm vibrotome sections were generated. The thick sections were put in Karnovsky's fixative (1% paraformaldehyde/3% glutaraldehyde/0.1 M sodium cacodylate buffer, pH 7.4) at 4°C overnight. Fixed tissue was then rinsed in water, post-fixed in reduced OsO_4_ (2% OsO_4_ + 1.5% potassium ferrocyanide, Sigma, prepared fresh) and stained en bloc with uranyl acetate before being dehydrated in ethanol, cleared in propyline oxide, and embedded in Eponate 12 (Ted Pella Co.). Thick sections were cut and stained with toludine blue then examined under a light microscope to select the area to be thin sectioned. Thin sections were cut by Leica Ultracut UCT microtome (Bannockburn, Il) and visualized using a Philips Tecnai 10 electron microscope (Eidhoven, Netherland). Images of cerebral blood vessels were captured with a SIA-L9C digital camera (Duluth, GA). For quantitative analysis, EC nuclei number, tight junction number, pericyte cell body/process length, and EC surface length were quantified using ImageJ software (NIH) on a minimum of six images of cerebral blood vessels per sample. To determine EC nuclei or tight junction number density, the EC nuclei or tight junction number was divided by the EC surface dimension as measured in microns. The percent coverage of EC surface by pericyte cell bodies was calculated by dividing the sum cell body/process length by the EC surface dimension.

### Pericyte and EC cell proliferation

To quantify Zic1+ pericyte nuclei density in the cortex at E14.5 and E18.5, the total number of Zic1+ nuclei was divided by the sum IB4+ vessel length in a 20× field. To determine pericyte cell proliferation at E14.5 and endothelial cell proliferation at E16.5, pregnant dams were injected with BrdU (50 mg/kg body weight) 2 hours prior to embryo collection. A pericyte labeling index was calculated by dividing the total number of BrdU+/Zic1+ pericytes by the total number of Zic1+ pericytes in a 20× field. All length measurements were performed using ImageJ software (NIH) on a minimum of 5, 20× fields per brain. For each genotype and age, the number of Zic1+ nuclei per vessel length and pericyte labeling index was calculated from samples collected from three separate litters (*n* = 3). Endothelial cell labeling index was quantified in the same manner except Lef-1 was used to label all ECs. The endothelial cell labeling index was calculated from control and mutant samples collected from 2 separate litters (*n* = 2).

### Quantitative analysis of vessel density/diameter and ICAM-1 expression

For all measurements, analysis was performed on E18.5 *PDGFrβ-cre*; *Foxc1fl-fl* mutants and littermate *PDGFrβ-cre*; *Foxc1fl-+* animals from three separate litters (*n* = 3). To determine mean vessel density, the sum length of IB4+ cerebral vessels was determined from a single 20× field. To determine mean vessel diameter, the average width of ∼20 IB4+ cerebral vessels per 20× field was calculated. To calculate the percentage of ICAM-1 expressing vessels, the sum length of ICAM-1+ vessels was divided by the sum length of IB4+ vessels (total vessel length) in a 20× field. All length, diameter and ICAM-1 measurements were performed using ImageJ software (NIH) on a minimum of five 20× fields per brain.

### BBB permeability

E18.5 *PDGFrβ-cre*; *Foxc1fl-fl* mutants and littermate controls (*PDGFrβ*; *Fox1fl-+* or *Foxc1fl-fl*) underwent transcardiac perfusion of Alexafluor-conjugated 555 cadaverine (200 µl of 0.25 mg/ml PBS; Invitrogen), biotin-conjugated 3 kD dextran, lysine fixable (3 ml of 0.15 mg/ml PBS; Invitrogen), or 70 kDa dextran, lysine fixable (3 ml of 3.5 mg/ml PBS; Invitrogen) followed immediately by 5 ml, 4% paraformaldehyde. Perfusions were performed by hand under a dissecting scope using 30 ½ gauge needles and a 1 or 5 ml syringe; care was taken to ensure that solutions were delivered at approximately the same rate for all animals. Following overnight fixation in 4% paraformaldehyde at 4°C, heads were processed through sucrose solutions and cryosectioned at 20 µm thickness. For animals injected for biotin-conjugate tracers, sections were washed in PBS then incubated in Alexafluor 488 streptaviden (1:500; Invitrogen) for 1 hour at room temperature. All sections were labeled with Alexafluor 633-conjugated isolectin-B4 (Invitrogen) to visualize blood vessels. For 3 kDa and 70 kDa tracers, fluorescent intensity in cortical tissue sections was determined using ImageJ software; images from *PDGFrβ*; *Foxc1fl-fl* mutants in which active hemorrhage was observed were not used in the quantitative analysis. Average fluorescent intensity of cerebral tissue for each animal was normalized to the fluorescent intensity of cerebral tissue sections from an animal perfused with 3 ml PBS followed by 5 ml 4% paraformaldehyde. For each tracer, a minimum of three mutants and three controls were used for qualitative (cadaverine) or quantitative (3 kDa and 70 kDa tracers) analysis (*n* = 3).

### Immunoblots

Cortices of E18.5 *PDGFrβ-cre*; *Foxc1fl-fl* mutants and littermate controls (*PDGFrβ-cre*; *Foxc1fl-+*, *Foxc1fl-fl* or *Foxc1fl-+*) from three separate litters (*n* = 3) were collected, lysed in RIPA buffer (Sigma–Aldrich) containing a protease inhibitor cocktail (Roche) and homogenized via sonication. Protein concentration for each sample lysate was determined using a Nanodrop and associated software (ThermoScientific). Lysates were combined with 4× sample buffer (300 mM Tris, 5% SDS, 50% glycerol, 0.025% bromophenol blue, 250 mM β-mercaptoethanol), run on Protean Tris-HCI 4–20**%** gradient gel (Bio-Rad) along with a Rainbow molecular weight standard (Amersham/GE), and transferred onto PVDF membranes (Bio-Rad). Immunoblots were blocked with 5% non-fat dehydrated milk (NFDM) for 1.5 hour then incubated overnight at 4°C in 2.5% NFDM containing one of the following primary antibodies: rat anti-glypican-3 (Novus), mouse anti-Par1 (BD Bioscience), goat anti-asporin (Novus), goat anti-MMP9 (R&D Systems). Following primary incubation, blots were washed then incubated in the 2.5% NFDM containing the appropriate horseradish peroxidase-linked secondary (1:5,000; Santa Cruz Biotechnology) for 45 min at room temperature. Clarity ECL substrate (Bio-Rad) and the ChemiDoc MP system (Bio-Rad) were used to visualize immunotagged protein bands. Densitometry of bands was performed using ImageLab software (Bio-Rad); density values were corrected for loading variations within each blot using the amount of β-actin expression.

### Microvessel isolation and multi-gene transcriptional profiling

For isolation of microvessels, E18.5 brains were dissected out, meninges removed and the forebrain region (cerebral cortex and striatum) was isolated in Hank's buffered salt solution (HBSS) and minced using a sharp forceps. Tissue was then incubated for 15 min at 37°C in HBSS containing 1% bovine serum albumin (BSA), 5 mg/ml Collegenase A, 10 mg/ml Dnase 1, and 5 mg/ml glucose. Following gentle pipetting to break up the tissue, the tissue suspension was passed through a 100 µm filter, pelleted via centrifugation (1,000 RPM for 5 min) then resuspended in HBSS containing 1% BSA. Cell suspension was then incubated with rat anti-PECAM-conjugated dynabeads (BD Bioscience) for 45 min at 4°C. Beads were collected using a magnetic particle concentrator followed by several washes with 1% BSA in HBSS. RNA was collected from bead-bound microvessels using the RNeasy Mini Kit (Qiagen). For expression of *Foxc1* transcript using RT-PCR, cDNA was generated from 100 ng of RNA using SuperScript® VILO™ cDNA Synthesis Kit (Invitrogen). For all other genes, multi-gene transcriptional profiling, a form of quantitative RT-PCR, was used to determine the number of mRNA copies per cell normalized to 18S rRNA abundance (10^6^ 18S-rRNA copies/cell) (Shih and Smith, 2005) (see supplementary material Table S1 for primer sequences). Multi-gene transcriptional profiling analysis was performed on microvessels isolated from 5 control (PDGFrβ-cre; Foxc1fl/+ or Foxc1fl/fl: *n* = 5) and 6 (*n* = 6) mutant E18.5 brains.

### Statistics

For pairwise analysis of control and mutant genotypes, Student *t*-tests were used. The standard error of the mean (SEM) is reported on all graphs.

## Supplementary Material

Supplementary Material
