# Supplementary material for: Foxc1 is required by pericytes during fetal brain angiogenesis
Source: Biol Open. 2013 May 20;2(7):647–59. doi: 10.1242/bio.20135009 (PMC3711032; doi:10.1242/bio.20135009)
Supplement: Supplementary Material [file supp_2_7_647__index.html]

Foxc1 is required by pericytes during fetal brain angiogenesis — Foxc1 is required by pericytes during fetal brain angiogenesis — Supplementary Material 

# Foxc1 is required by pericytes during fetal brain angiogenesis

## bio.20135009 Supplementary Material

**Files in this Data Supplement:**

- Supplementary Material - Julie A. Siegenthaler et al. doi: 10.1242/bio.20135009
