# Supplementary material for: Foxc1 is required by pericytes during fetal brain angiogenesis
Source: Biol Open. 2013 May 20;2(7):647–59. doi: 10.1242/bio.20135009 (PMC3711032; doi:10.1242/bio.20135009)
Supplement: Supplementary Material [file supp_bio.20135009_bio.20135009-s1.pdf]

Supplementary Material

Julie A. Siegenthaler et al. doi: 10.1242/bio.20135009

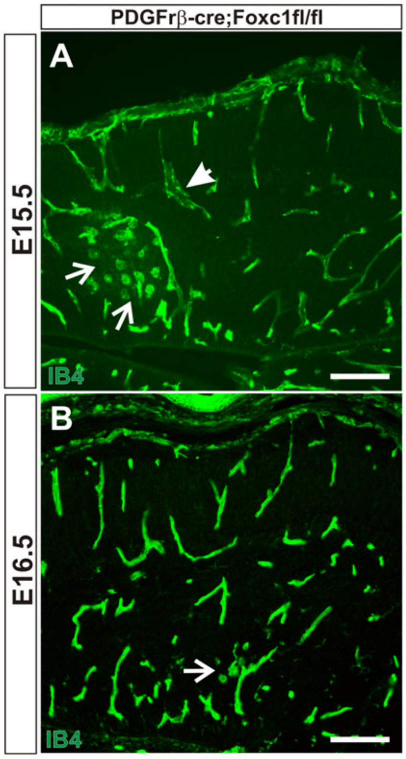

Fig. S1. Vascular defects at earlier developmental stages in pericyte conditional Foxc1 mutants. Isolectin-B4 staining in E15.5 (**A**) and E16.5 (**B**) PDGFrβ-cre; Foxc1fl-fl mutants reveals dysplastic vessels (arrows in A) and activated microglia with amoeboid morphology (arrowheads in A,B) indicative of active inflammation caused by hemorrhage. Scale bars: 100 μm.

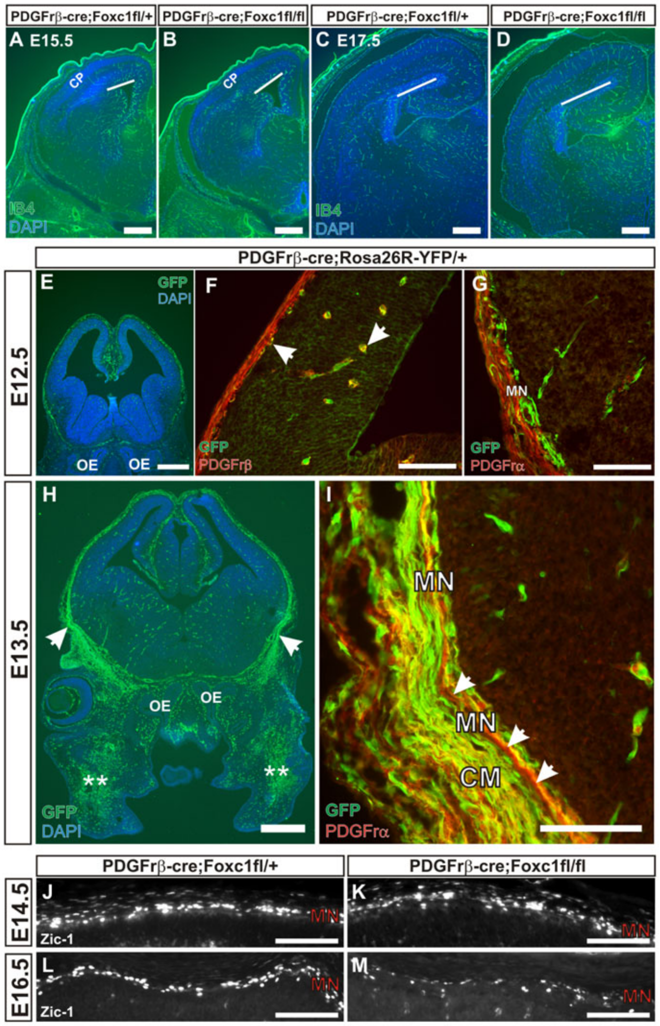

Fig. S2. Cortical and meningeal phenotype in PDGFrβ-cre; Foxc1fl-fl mutants. (**A–D**) Low-magnification of E15.5 (A,B) and E17.5 (C,D) PDGFrβ-cre; Foxc1fl-+ and PDGFrβ-cre; Foxc1fl-fl mutants labeled with IB4 and DAPI. White lines represent the length of the dorsal cortical neuroepithelium, which is longer in PDGFrβ-cre; Foxc1fl-fl mutants. (**E**) Low-magnification image of E12.5 PDGFrβ-cre; Rosa26-YFP/+ head immunolabeled with an anti-GFP antibody shows signal in perineural vascular plexus (PNVP) encircling the brain and associated with blood vessels both within the brain and in the head tissue. (**F**,**G**) E12.5 PDGFrβ-cre; Rosa26-YFP/+ brain and adjacent PNVP and meninges/mesenchyme with GFP and PDGFrβ (F) or PDGFrα (G) immunolabeling. In F, PDGFrβ/GFP co-labeled cells are observed in the brain vasculature and in the adjacent PNVP (arrows) corresponding to pericytes. Note that PDGFrβ+ (F) and PDGFrα+ (G) meninges/mesenchyme outside the PNVP is not immunopositive for GFP. (**H**) Low-magnification image of E13.5 PDGFrβ-cre; Rosa26-YFP/+ head immunolabeled with an anti-GFP antibody shows recombination in the brain and head vasculature but also in the calvarial mesenchyme (arrows) and portions of the facial mesenchyme (asterisks). (**I**) E13.5 PDGFrβ-cre; Rosa26-YFP/+ brain and adjacent PNVP, meninges, calvarial mesenchyme with GFP and PDGFrα dual-immunolabeling. There is extensive co-labeling of GFP and PDGFrα in the calvarial mesenchyme (CM). In the meninges (MN), GFP/PDGFrα co-labeling is evident but some cells in the meningeal layers are not GFP+ (arrows). (**J–M**) PDGFrβ-cre; Foxc1fl-+ and PDGFrβ-cre; Foxc1fl-fl mutants at E14.5 (J,K) and E16.5 (L,M) with Zic-1 immunolabeling to visualize cell density in the dorsal cortical meninges (MN). Scale bars: 500 μm (A–E,H); 100 μm (F,G,I–M). OE: olfactory epithelium. PNVP: perineural vascular plexus.

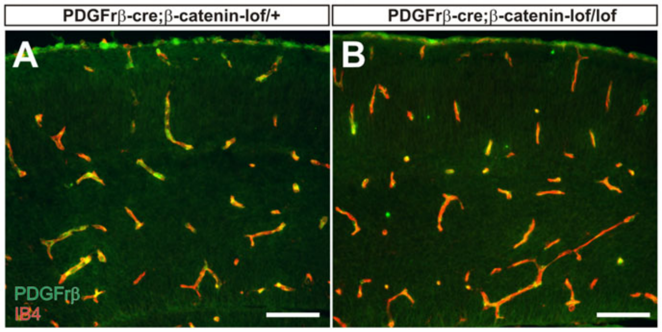

Fig. S3. Normal cerebral vasculature in PDGFrβ-cre; β-catenin-lof-lof mutant. (**A**,**B**) PDGFrβ (green) and IB4 (red) immunostaining of PDGFrβ-cre; β-catenin-LOF/+ and PDGFrβ-cre; β-catenin-lof/lof mutant cerebral cortices at E17.5. Scale bars: 100 μm.

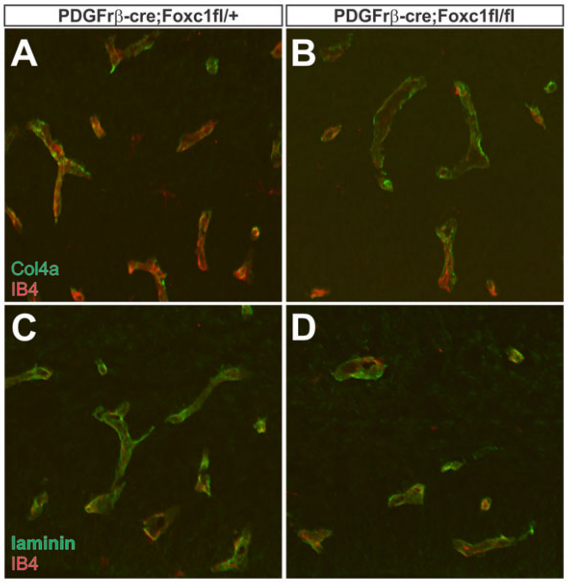

Fig. S4. ECM immunolabeling at E16.5. (**A**,**B**) Col4a (green) immunolabeling in the cerebral cortex of a PDGFrβ-cre; Foxc1fl-+ control and a PDGFrβ-cre; Foxc1fl-fl mutant surrounds IB4+ (red) blood vessels to a similar extent. (**C**,**D**) Laminin (green) immunolabeling coats cerebral blood vessels to a similar degree in both control and mutant samples.

Table S1.

Gene	Forward primer	Reverse primer
Gpc3	CTACCATCCATGATTCCATCCAGTA	CTGGGAGTGGGCACACAAC
Col3a1	AAGCTGTTGCCTCTACTCAATTCA	CTTGGATGCCAGTCTGACTTTG
Col1a2	CACCCCAGCGAAGAACTCATAC	TCAAACTGGCTGCCACCAT
Col4a2	CCCATCTGACATCACACTTGTTG	TGAGATTACGCCGGGTATCC
Col6a3	CAGGGCCATCTACCAAGGAA	CTCGGTGTGCTGCCTGAAG
Lama2	TGGTGAAGGCTATGCATTAGTGA	TCCGGAACTTGAACATGACTGT
Lamc3	AAGAGGCAGCCAAGCTGAAG	GGGCTACCACAGTCAGACCTTT
Vtn	GGCTGACCAAGAGTCATGCA	TCGTCACACTGACACTTCTTGCT
TGFβ1	GCTGCTGACCCCCACTGAT	TGTATTCCGTCTCCTTGGTTCA
TGFβr2	CAGCATCACGGCCATCTGT	CCGTCTCCAGAGTAATGTTCTTGTC
p21	GTGATTGCGATGCGCTCAT	GGCGTCTCCGTGACGAAGT
p27	CTTCCGCCTGCAGAAACCT	CTCGGCAGTGCTTCTCCAA
Cyclin D1	CGCCCTCCGTATCTTACTTCAA	CTCACAGACCTCCAGCATCCA
Angpt2	GCTGAAGGACTGGGAAGGC	GGACTCTTCACCAGCGAGGTA
Angpt1	CATTCTTCGCTGCCATTCTG	GCACATTGCCCATGTTGAATC
Edn1	GGCCACAGACCAGGCAGTT	GGTACTTTGGGCCCTGAGTTC
Esm1	CCCACACAGAGCGTGACTCA	AGCGTTCCCTTCTCCAATCTC
Ptgs2	GTGTCCCTTCACTTCTTTCAATGTG	TCTGGAGTGGGAGGCACTTG
PDGFrβ	CTGTGAATGCCGTGCAGACT	TGGAAGTTCACCACATCATTGC
CD144	CAACTTCACCCTCATAAACAACCAT	ACTTGGCATGCTCCCGATT
CD41	GTGATGGCCACTGTCCAACTG	GTGATGGCCACTGTCCAACTG
F2R (Par1)	GAAGTCGCGGGCTTTGTTC	GCCCAAAGCAGACGATGAAG
MMP9	CAGACGTGGGTCGATTCCA	TCGCGGCAAGTCTTCAGAGT
Notch3	GATGGCTCTACTGCACTGATCCT	TGTAAGGCAGATTTCCCAAGCT
S1pr3	GCCTAGCGGGAGAGAAACCT	CCGACTGCGGGAAGAGTGT
Itgb5	CCAATGGGAGGTCCAACTTG	TCAGGAGGATGCTGCCAAC
Cspg4 (Ng2)	GGCCCTTATCTTGCCTCTGC	GCGGTCAACACCTGGACATC
Connexin4	CTCTGTGATGTACCTGGGATATGC	CTCCGAGCTGCCTTCTTGTC
BMP5	CACAGACCCAGGCCCTTTT	CTCCGACTCCTCAGGGTTGTC
Dlk1	TTCGGCCACAGCACCTATG	TGGCACCTGCAGACATTGTC
Angptl2	GCATCGTGAGCGAGGTGAA	TGCATGTACAGCTGCGTGACT
Aspn	CAAGGGAATGAATGCTTTACATGT	CCTTCAAATGCCCCTGGTT
